# Divergent expression patterns of *SATB1* mRNA and SATB1 protein in colorectal cancer and normal tissues

**DOI:** 10.1007/s13277-015-3084-0

**Published:** 2015-01-21

**Authors:** Anna E. Kowalczyk, Janusz Godlewski, Bartlomiej E. Krazinski, Jolanta Kiewisz, Agnieszka Sliwinska-Jewsiewicka, Przemyslaw Kwiatkowski, Bartosz Pula, Piotr Dziegiel, Jacek Janiszewski, Piotr M. Wierzbicki, Zbigniew Kmiec

**Affiliations:** 1 Department of Human Histology and Embryology, Faculty of Medical Sciences, University of Warmia and Mazury, 30 Warszawska Str., 10082 Olsztyn, Poland; 2 Department of Histology and Embryology, Wroclaw Medical University, 50368 Wroclaw, Poland; 3 Department of Physiotherapy, Wroclaw University School of Physical Education, 51617 Wroclaw, Poland; 4 Department of Internal Medicine, Gastroenterology, Cardiology and Infectionology, Faculty of Medical Sciences, University of Warmia and Mazury, 10082 Olsztyn, Poland; 5 Department of Histology, Medical University of Gdansk, 80210 Gdansk, Poland

**Keywords:** *SATB1* expression, Colorectal cancer, Normal colonic mucosa, Survival

## Abstract

Special AT-rich sequence-binding protein 1 (SATB1) is a ‘genome organizer,’ and it has been proposed as a factor that affects the development and progression of various human neoplasms, including colorectal cancer (CRC). This study aimed to compare SATB1 expression in a group of CRC patients and healthy subjects at the mRNA and protein levels. We collected paired tumor tissue and unchanged mucosa of the large intestine from 102 CRC patients as well as 53 biopsies of normal colon mucosa obtained from healthy patients during screening colonoscopy. Tissue samples were quantified for *SATB1* mRNA by quantitative PCR, while SATB1 protein expression was determined by Western blotting and immunohistochemistry. *SATB1* mRNA level in tumor tissues was over twofolds lower than in samples of corresponding unchanged tissues and fourfolds lower than in biopsies of healthy colon mucosa. Western blotting analysis revealed that SATB1 protein content in tumor and unchanged tissues of CRC patients was over sixfold and fivefolds higher than in biopsies of healthy colon mucosa, respectively. Immunohistochemical staining demonstrated higher nuclear and cytoplasmic SATB1 reactivity in the tumor tissue compared to unchanged mucosa of CRC patients. Despite these differences, *SATB1* mRNA, protein, and immunoreactivity levels did not correlate with patients’ clinicopathological data and their overall survival, but the latter analysis was limited by a relatively short period of follow-up. In conclusion, we suggest that some as yet unidentified posttranscriptional mechanisms that regulate SATB1 expression may be altered in the CRC tissue.

## Introduction

Colorectal cancer (CRC) is the third most commonly diagnosed cancer accounting for about 10 % of total adult malignancies worldwide. In 2008, there were approximately 1.2 million cases of newly diagnosed CRC and over 600,000 people died of this malignancy [1]. A large part of the malignancies are diagnosed at the late stage, and thus, the major cause of death in individuals suffering from CRC is distant metastasis. Death from CRC can be prevented by the detection of early-stage disease. Over the past decades, a molecular background of CRC pathogenesis has been extensively screened for potential molecular markers and therapeutic targets. Studies suggest a number of factors which can influence the development of the CRC including genetic factors. Gene activation or silencing can be regulated by changes in chromatin organization. Special AT-rich sequence-binding protein 1 (SATB1) is a nuclear matrix-associated protein which organizes the structure of genome at the chromatin level. SATB1 forms a docking site for the chromatin-modifying enzymes and transcription activators or repressors and, as a potent epigenetic regulator, may affect the transcription of numerous genes [2]. SATB1 can influence the expression of more than 1000 genes, including those implicated in the pathogenesis of human neoplasms [3]. This protein may play a role in breast cancer cell proliferation [4] and was found to be upregulated in several malignancies such as breast, laryngeal, gastric, liver, and ovarian cancers [3, 5–8]. Results of many studies suggest that SATB1 overexpression is associated with an aggressive phenotype of tumor cells. In breast cancer, SATB1 was found to directly upregulate metastasis-associated genes while it decreased expression of tumor-suppressor genes and promoted tumor growth and metastasis [3]. Silencing of SATB1 expression in breast cancer cell lines restored normal acinar polarity and limited the ability of cells to grow and metastasize in vivo. Moreover, ectopic SATB1 expression in a nonaggressive breast cancer cell line induced the aggressive phenotype and metastatic activity in the cells [3]. SATB1 overexpression can affect proteins mediating cell-to-cell adhesion and promote epithelial-mesenchymal transition (EMT) [3]. Altered SATB1 expression could be related to the occurrence and development of multidrug resistance phenotype in breast cancer [9]. In some studies, the expression level of SATB1 correlated with cancer progression and was suggested to be an useful prognostic marker in breast cancer, laryngeal squamous cell carcinoma, cutaneous melanoma, glioma, gastric, and hepatocellular cancer [3, 5–7, 10]. Several discrepancies considering the role of SATB1 in human malignancies have been also documented. Iorns et al. [11] concluded that SATB1 had no role in breast cancer pathogenesis, contradicting the results of the abovementioned study [3]. In one study of non-small cell lung cancer, the loss of SATB1 expression was associated with poor survival [12], while in another study of the same cancer type, the opposite relationship was proposed, demonstrating the highest level of *SATB1* mRNA in metastatic cancers [13]. Altogether, the reports suggest that *SATB1* can be expressed in a tissue-typical manner, and prognostic value of SATB1 may be cancer-type specific; however, contradictory results could be observed even in the same tumor type.

*SATB1* expression levels have been examined in tumor and unchanged tissue samples of patients suffering from rectal or colorectal cancer [14–18], but so far, there was no comparison of the level of *SATB1* expression in CRC tissue and normal colon mucosa of healthy subjects. Moreover, in some aspects, as the difference in the level of *SATB1* expression in CRC tissue and its prognostic significance, the results of mentioned studies were inconsistent. Therefore, the main objective of our study was to analyze and compare *SATB1* gene expression in samples of tumor and unchanged colorectal tissues of CRC patients as well as in mucosal colon biopsies in a group of healthy subjects. Quantitative real-time PCR and Western blotting techniques were used to assess *SATB1* mRNA and SATB1 protein levels, respectively. An immunohistochemical detection of SATB1 protein was carried out to localize its expression pattern within the tested tissues of CRC patients.

## Materials and methods

### Patients

The specimens were obtained from two Polish surgical clinics (Hospital of the Ministry of Internal Affairs and Administration in Olsztyn and Hospital of the Medical University of Gdansk) and the gastrointestinal endoscopic unit (Hospital of the Ministry of Internal Affairs and Administration in Olsztyn) from 2010 to 2012. The study included 102 patients with CRC (demographic and clinicopathological data are presented in Table 1). None of the CRC patients had a second neoplastic disease or had previously undergone chemo- or radiotherapy. The control group consisted of 53 healthy individuals (14 males and 39 females, average age 57.3 ± 6.83 years, range 36–82 years; mean ± SD) who underwent colonoscopy as part of routine surveillance for CRC (within the National screening program for early detection of colorectal cancer). Control subjects had no family history of CRC. None of the CRC patients or healthy individuals suffered from inflammatory bowel disease. Clinical and demographic data were obtained at the time of enrollment. Median follow-up time was 36.2 months. Written informed consent was obtained from each patient included in the study. All procedures were performed in accordance with the ethical standards and were approved by the Bioethics Committee of the University of Warmia and Mazury in Olsztyn.

**Table 1:** Demographic and clinicopathological characteristics of studied CRC patients

Parameter	Number of cases	Percentage (%)
Total	102	100.0
Sex		
Male	54	52.9
Female	48	47.1
Age (years)		
≤67	49	48.0
>67	53	52.0
Localization		
Cecum, ascending, and transverse colon	37	36.3
Descending and sigmoid colon	26	25.5
Rectum	39	38.2
Depth of invasion (pT status)		
T1	1	1.0
T2	13	12.7
T3	70	68.6
T4	18	17.6
Lymph nodes (pN status)		
N0	55	53.9
N1	30	29.4
N2	17	16.7
Metastasis (pM status)		
M0	87	85.3
M1	15	14.7
TNM stage		
I	13	12.7
II	38	37.3
III	36	35.3
IV	15	14.7

### Collection of colorectal samples

All steps of material collection were standardized in all collaborative clinics. CRC samples were obtained during surgical hemicolectomy, and control group specimens were collected during colonoscopy. In CRC patients’ group, two types of matched samples were taken within 20 min after tumor resection: (i) tumor tissue and (ii) macroscopically unchanged mucosa from a distant part of resected large intestine. Specimens were immediately cut in two samples (5 × 5 × 5 mm) for quantitative PCR (qPCR) and Western blotting (WB) analyses, frozen in liquid nitrogen, and stored at −80 °C, whereas for routine histological evaluation and immunohistochemistry (IHC), the samples were fixed in 10 % neutral buffered formalin and further processed into paraffin blocks. In the control group of healthy patients, one biopsy (2 × 2 × 2 mm) was fixed in 10 % neutral buffered formalin for routine histological examination, and two specimens from the adjacent location to the biopsy site were collected for qPCR or WB assays. Colonoscopic biopsies for qPCR analysis were immediately placed in sterile vials containing RNAlater (Sigma-Aldrich, St. Louis, MO, USA) and stored at −20 °C until further analysis, while for WB assay, the procedure was the same as for CRC samples.

### Total RNA extraction and reverse transcription

Total RNA was extracted from paired samples of cancer tissue and unchanged mucosa derived from 69 CRC patients and 36 colonoscopic biopsies of healthy subjects using a Total RNA Prep Plus kit (A&A Biotechnology, Gdynia, Poland), according to the procedure provided. Isolated RNA was quantified with spectrophotometry (NanoDrop 1000, NanoDrop products, Wilmington, DE, USA). Reverse transcription was carried out in a vial containing 20 μl reaction mixture of 2 μg of total RNA, 0.5 μg of oligo dT primers (Sigma-Aldrich), 200 U of RevertAid™ Reverse Transcriptase, 20 U of RiboLock™ RNase Inhibitor, and 1 mM of each dNTP (all Thermo Scientific, Waltham, MA, USA). Reactions were conducted according to the manufacturer’s instructions. Resulting cDNAs were diluted sixfold with sterile water and stored at −20 °C until further use.

### Real-time quantitative PCR

Quantification of *SATB1* gene expression was carried out using ABI 7500/7500 Fast Real-Time PCR System (Life Technologies-Applied Biosystems, Foster City, CA, USA). β-Actin (*ACTB*) and hypoxanthine phosphoribosyltransferase 1 (*HPRT1*) genes were used as an internal control to normalize the transcript levels of *SATB1*. qPCR conditions were validated and showed 90–100 % efficiency for all assays. The amplification primer pairs were 5′-AGCAGGAAATGAAGCGTGCTAAAG-3′ and 5′-GATCATGGAGAGGTTCTCCCACAG-3′ for *SATB1*, 5′-TGTGCCCATCTACGAGGGGTATGC-3′ and 5′-GGTACATGGTGGTGCCGCCAGACA-3′ for *ACTB*, 5′-GACTTTGCTTTCCTTGGTCAGGC-3′ and 5′-TGGCGATGTCAATAGGACTCCAG-3′ for *HPRT1*. The reaction mixture (20 μl) included 2.4 μl of cDNA, 0.2 μmol/l each of forward and reverse primers, and 10 μl of Fast SYBR® Green PCR Master Mix (Life Technologies—Applied Biosystems). The following general qPCR conditions were applied: initial denaturation for 20 s at 95 °C, followed by 40 cycles of denaturation at 95 °C for 3 s, and annealing and elongation at 58 °C for 30 s (*SATB1*), 60 °C for 40 s (*ACTB*), or 62 °C for 40 s (*HPRT1*). All samples were amplified in duplicates. Standard curves consisting of serial dilutions of the appropriate cDNA were used to control the efficiency of qPCR reactions. After qPCR, melting curves were obtained by stepwise increases in temperature from 60 to 95 °C to ensure that a single product was amplified in the reaction. No template control reactions were performed for each qPCR run. Selected PCR products were subjected to gel electrophoresis which confirmed their predicted size. Relative quantification of *SATB1* expression was evaluated using the ΔΔCt method [19]. The fold change in the relative *SATB1* gene expression was determined by calculating the 2^−ΔΔCt^ value. Fold increase above 1 (2^−ΔΔCt^ >1) indicated *SATB1* overexpression in CRC and fold decrease under 1 (2^−ΔΔCt^ <1) indicated *SATB1* downregulation.

### Protein isolation and Western blotting analysis

Paired samples of tumor tissue and unchanged mucosa derived from 32 CRC patients and 17 colonoscopic biopsies were homogenized in RIPA lysis buffer supplied with 1:100 protease inhibitor cocktail, 1:100 phosphatase inhibitor cocktail 2, and 5 mM EDTA (all Sigma-Aldrich). Homogenates were briefly centrifuged to remove tissue debris. Then, samples were centrifuged twice at 9000*g* for 10 min at 4 °C. The protein content in the supernatant was determined by the Bradford method [20]. Samples were aliquoted and stored at −80 °C until further use.

To determine the level of SATB1 protein in tissue lysates, the SDS-PAGE followed by Western blotting assays were performed. Isolated protein fractions were denatured for 5 min at 95 °C and loaded on polyacrylamide gel (30 μg/lane). Molecular weight standard (Spectra Multicolor Broad Range Protein Ladder, Thermo Scientific) and protein extracts from human tonsils (a positive control) were included into each blotting experiment. Gels were run at the 10 mA/gel during migration in the stacking gel and 15 mA/gel in the separating gel (10 %). Proteins were transferred onto PVDF membrane (Western blot membrane, Roche, Mannheim, Germany). Blots were blocked in 5 % nonfat dry milk dissolved in Tris-buffered saline pH 7.5 with 0.1 % Tween-20 (TBS-T) followed by overnight incubation at 4 °C with a primary rabbit polyclonal anti-human SATB1 antibody (diluted 1:1000 in TBS-T buffer; #3650, Cell Signaling Technology, Danvers, MA, USA). A rabbit polyclonal anti-human actin antibody (diluted 1:100 in TBS-T; #A2066, Sigma-Aldrich) was used to confirm equal loading of proteins. Primary antibodies were washed out with TBS-T. The membranes were treated with the specific HPR-conjugated goat anti-rabbit IgG secondary antibodies (diluted 1:5000 in TBS-T; #A0545, Sigma-Aldrich) for 90 min at room temperature (RT), developed with an enhanced chemiluminescence (SuperSignal West Pico Chemiluminescent Substrate, Thermo Scientific), and visualized with G:BOX iChemi XR imaging system (Syngene, Cambridge, UK). Band intensity was quantified using ImageJ software (NIH, Bethesda, MD, USA). Positive SATB1 control—human tonsil extract—was used to normalize SATB1 quantities between individual blotting experiments. For the negative control, SATB1 antibody was omitted and substituted with phosphate-buffered saline (PBS). Optical density (OD) ratios between tumor and the corresponding unchanged tissue of CRC patients were calculated. The ratios higher than 1 indicated that the expression of SATB1 protein was upregulated in CRC while those lower than 1 were regarded as downregulated.

### Immunohistochemical staining

SATB1 immunoreactivity was analyzed in 102 tumor and 39 unchanged large-intestine tissues sampled from CRC patients. Immunohistochemistry was performed on 4-μm-thick paraffin sections in Autostainer Link48 (DakoCytomation, Glostrup, Denmark) to ensure repeatable reaction conditions. The sections were first boiled in Target Retrieval Solution buffer (pH 9) using a Pretreament Link Platform (both DakoCytomation) in order to retrieve the antigens. The sections were then cooled in a rinsing buffer (TBS/0.05 % Tween) and endogenous peroxidase activity was blocked by incubation with EnVision FLEX Peroxidase-Blocking Reagent (DakoCytomation). After rinsing the sections with TBS/0.1 % Tween, rabbit monoclonal primary antibody directed against SATB1 (1:100, EPR3951, GeneTex, Irvine, CA, USA) was applied for 20 min at RT. Sections were then washed in TBS/0.05 % Tween and EnVision FLEX reagent was applied in accordance with the manufacturer’s instructions to visualize the studied antigens. After washing the sections in TBS/0.05 % Tween, EnVision FLEX/HRP secondary antibody was applied (20 min at RT). In the next step, the substrate for peroxidase, diaminobenzidine, was applied, and the sections were incubated for 10 min at RT. Last, the sections were counterstained with Mayer’s hematoxylin, dehydrated in alcohols (70, 96, 99.8 %) and xylene, and finally mounted using the SUB-X Mounting Medium (DakoCytomation). As positive immunoreactive control for the SATB1, a CRC specimen previously characterized by high SATB1 expression in cancer cells and lymphocytes was used, whereas the negative controls were performed by omitting the primary antibody.

### Evaluation of immunohistochemical reactions

The SATB1 immunostained sections were evaluated using Olympus BX53 light microscope (Olympus, Tokyo, Japan) by two independent pathologists who were blinded to the patients’ clinical data. In doubtful cases, reevaluation was performed until a consensus was achieved. SATB1 immunoexpression was assessed in whole tissue sections in cancer cells and non-transformed, normal cells of the mucosa according to the immunoreactive score (IRS) of Remmele and Stegner [21]. The scale is based on the percentage of cells showing positive reaction (0 points: absence of cells with positive reaction, 1 point: 1–10 % cells, 2 points: 11–50 %, 3 points: 51–80 %, 4 points: over 80 % cells with positive reaction), as well as reaction intensity (0: no reaction, 1: low intensity reaction, 2: moderate intensity reaction, 3: intense reaction). The final score is the product of both parameters and ranges from 0 to 12 points. The IRS method was used for separate assessment of both nuclear as well as cytoplasmic SATB1 expression in enterocytes and cancer cells. Based on median expression values, CRC cases which showed a nuclear SATB1 expression less than or equal to four IRS scores were regarded as having ‘low’ expression, whereas those scored more than four were regarded as ‘high’ SATB1 expression. In the case of cytoplasmic SATB1 immunoreactivity, cases showing no expression were regarded as ‘negative’ and those scoring one or more were regarded as ‘positive’.

### Statistical analyses

Statistical analyses were performed using Prism 5 (GraphPad, La Jolla, CA, USA) and STATISTICA v.10 (StatSoft, Tulsa, OK, USA) software. The differences in *SATB1* mRNA and SATB1 protein levels between matched tumor and unchanged samples of CRC patients were examined by the Wilcoxon matched-pairs test, whereas differences between colon mucosa biopsies of healthy subjects and tissues of CRC patients were assessed by Mann-Whitney *U* test. SATB1 immunoexpression was analyzed utilizing the Mann-Whitney *U* test and Wilcoxon matched-pairs test. The correlations between the demographic, clinicopathological, and molecular parameters were analyzed by the Fisher’s exact test. Survival curves were plotted using Kaplan-Meier method. The statistical significance of differences in survival between groups of patients based on various variables (level of *SATB1* expression and demographic and clinicopathological characteristics) was evaluated using the log-rank test and Cox regression method. In all the analyses, results were considered statistically significant when *P* < 0.05.

## Results

### *SATB1* mRNA expression in CRC tissues is downregulated

**Fig. 1:**
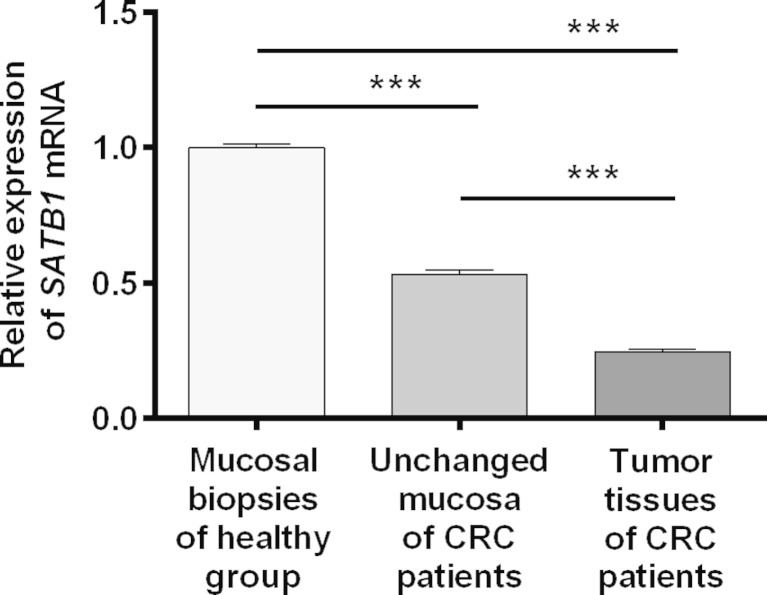
*SATB1* mRNA levels in the tumor and unchanged large-intestine tissues of CRC patients and normal colon mucosa of healthy subjects. Expression of *SATB1* mRNA (mean ± SEM) is shown in relation to the value obtained for healthy controls (1.0). ***P < 0.001

To determine the expression of *SATB1* at the mRNA level, matched tumor and unchanged tissues derived from CRC patients and colonic biopsies of healthy group were subjected to qPCR analysis. *SATB1* mRNA was found in all studied tissue samples of CRC patients and colonic biopsies of healthy individuals. The expression of *SATB1* mRNA in healthy colon mucosa and unchanged tissue of CRC patients was significantly elevated when compared to the tumor tissues (1.00 ± 0.01 and 0.53 ± 0.01 vs. 0.25 ± 0.01, respectively; *P* < 0.0001; Fig. 1). *SATB1* mRNA level was higher in the colon mucosa of healthy individuals than in unchanged tissues of CRC patients (1.00 ± 0.01 vs. 0.53 ± 0.01; *P* < 0.0001; Fig. 1). Among the 69 tumor specimens tested, the relative *SATB*1 mRNA level (tumor tissue vs. matching unchanged mucosa of CRC patients) was decreased in 51 (73.9 %) tumors while it was increased in 18 (26.1 %) cases (Table 2).

**Table 2:** Associations between clinicopathological features of studied CRC patients and the relative mRNA expression of *SATB1* in colorectal tumor tissues

Parameter	Number of cases	*SATB1* mRNA levels in tumor vs. unchanged tissues of CRC patients					
		Percentage (%)	Down (ratio <1)	Percentage (%)	Up (ratio >1)	Percentage (%)	*P* values
Total	69	100.0	51	73.9	18	26.1	
Sex							
Male	35	50.7	29	82.9	6	17.1	0.1055
Female	34	49.3	22	64.7	12	35.3	
Age (years)							
≤67	31	44.9	21	67.7	10	32.3	0.4092
>67	38	55.1	30	78.9	8	21.1	
Localization							
Cecum, ascending, and transverse colon	26	37.7	17	65.4	9	34.6	0.4081
Descending and sigmoid colon	16	23.2	12	75.0	4	25.0	
Rectum	27	39.1	22	81.5	5	18.5	
Depth of invasion (pT status)							
T1 + T2	8	11.6	7	87.5	1	12.5	0.6705
T3 + T4	61	88.4	44	72.1	17	27.9	
Lymph nodes (pN status)							
N0	36	52.2	25	69.4	11	30.6	0.4219
N1 + N2	33	47.8	26	78.8	7	21.2	
Metastasis (pM status)							
M0	57	82.6	43	75.4	14	24.6	0.4966
M1	12	17.4	8	66.7	4	33.3	
TNM stage							
I + II	32	46.4	23	71.9	9	28.1	0.7874
III + IV	37	53.6	28	75.7	9	24.3	

### The lack of correlation between *SATB1* mRNA expression and clinicopathological features

To assess the impact of *SATB1* expression at the mRNA level on CRC pathogenesis, the relationships between *SATB1* mRNA content and selected demographic and clinicopathological parameters were tested. The relative *SATB1* mRNA level did not correlate with patients’ sex, age, tumor localization, TNM disease stage, depth of invasion, lymph node involvement, and the presence of metastases (Table 2).

For the reason that the groups of CRC patients and healthy controls differed in terms of gender ratio and mean age, the possible correlations between these parameters and the expression levels of *SATB1* mRNA had to be excluded. Pearson correlation and Mann-Whitney *U* test proved the lack of significant relationships (*P* > 0.05; data not shown); and therefore, we assumed that this diversity had no impact on the performed analyses.

### SATB1 protein content is elevated in tissues of CRC patients

**Fig. 2:**
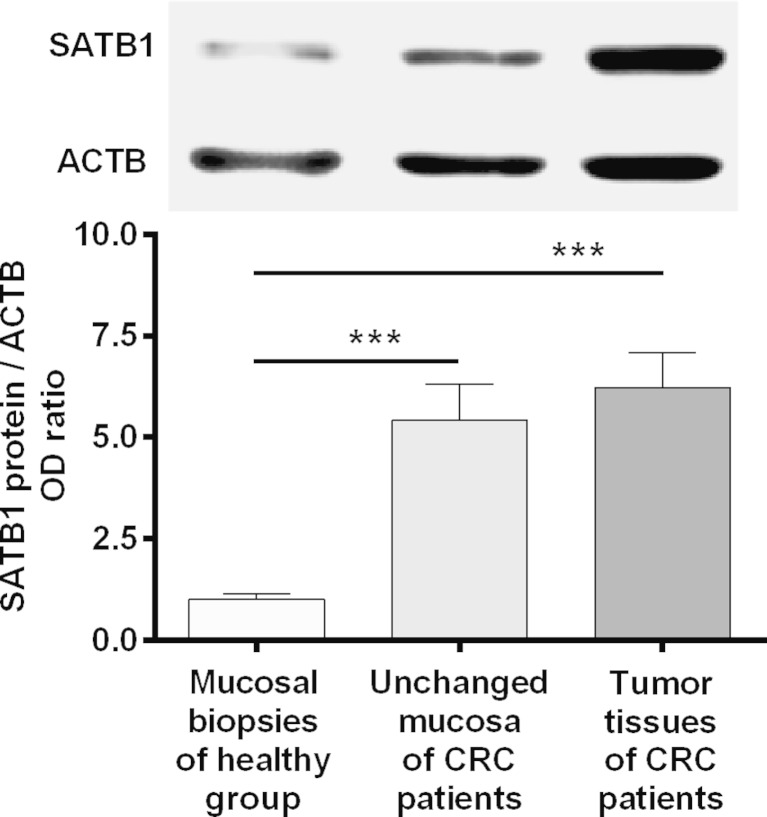
SATB1 protein levels in the tumor and unchanged large-intestine tissues of CRC patients and normal colon mucosa of healthy subjects. Representative blots of studied tissues are shown above the graph. β-Actin (ACTB) was used as loading control. *Bars* represent mean ± SEM. *** *P* < 0.001

To determine the expression of SATB1 at the protein level, matched tumor and unchanged tissues derived from CRC patients and colonic biopsies of healthy group were subjected to Western blotting analysis. SATB1 protein was found in all studied tissues of CRC patients and colon mucosa of healthy subjects. SATB1 protein content in both tumor and unchanged tissue samples of CRC patients was significantly higher than the amount of SATB1 protein in colonic biopsies of the healthy group (6.23 ± 0.86 and 5.42 ± 0.89 vs. 1.00 ± 0.13, respectively; *P* < 0.0001; Fig. 2). The average content of SATB1 protein in tumor tissue was similar to that in unchanged mucosa of the large intestine of CRC patients (6.23 ± 0.86 vs. 5.42 ± 0.89, respectively; *P* = 0.56; Fig. 2). Among 32 tumor tissue specimens tested, the relative content of SATB1 protein (tumor tissue vs. matching unchanged mucosa of CRC patients) was downregulated in 15 (46.9 %) tumors while it was upregulated in 17 (53.1 %) cases (Table 3).

**Table 3:** Associations between clinicopathological features of studied CRC patients and the relative SATB1 protein levels (assessed by Western blotting) in colorectal tumor tissues

Parameter	Number of cases	Percentage (%)	SATB1 protein levels in tumor vs. unchanged tissues of CRC patients				*P* values
			Down (ratio <1)	Percentage (%)	Up (ratio >1)	Percentage (%)	
Total	32	100.0	15	46.9	17	53.1	
Sex							
Male	15	46.9	5	33.3	10	66.7	0.1777
Female	17	53.1	10	58.8	7	41.2	
Age (years)							
≤67	14	43.8	5	35.7	9	64.3	0.3075
>67	18	56.3	10	55.6	8	44.4	
Localization							
Cecum, ascending, and transverse colon	14	43.8	9	64.3	5	35.7	0.0954
Descending and sigmoid colon	7	21.9	1	14.3	6	85.7	
Rectum	11	34.4	5	45.5	6	54.5	
Depth of invasion (pT status)							
T1 + T2	7	21.9	3	42.9	4	57.1	1.0000
T3 + T4	25	78.1	12	48.0	13	52.0	
Lymph nodes (pN status)							
N0	23	71.9	10	43.5	13	56.5	0.6989
N1 + N2	9	28.1	5	55.6	4	44.4	
Metastasis (pM status)							
M0	29	90.6	13	44.8	16	55.2	0.5887
M1	3	9.4	2	66.7	1	33.3	
TNM stage							
I + II	22	68.8	10	45.5	12	54.5	1.0000
III + IV	10	31.3	5	50.0	5	50.0	

For the reason that the groups of CRC patients and healthy controls differed in terms of gender ratio and mean age, the possible correlations between these parameters and the levels of SATB1 protein had to be excluded. Pearson correlation and Mann-Whitney *U* test proved the lack of significant relationships (*P* > 0.05; data not shown), and therefore, we assumed that this diversity had no impact on the performed analyses.

### Nuclear and cytoplasmic SATB1 immunoreactivity is elevated in CRC tissues

Immunohistochemical examination of SATB1 expression included two sub-analyses: total 102 tumor cases vs. 39 unchanged colon tissues of CRC patients (Fig. 4a, b) and matched tumor vs. normal colon samples from 39 CRC patients.

**Fig. 3:**
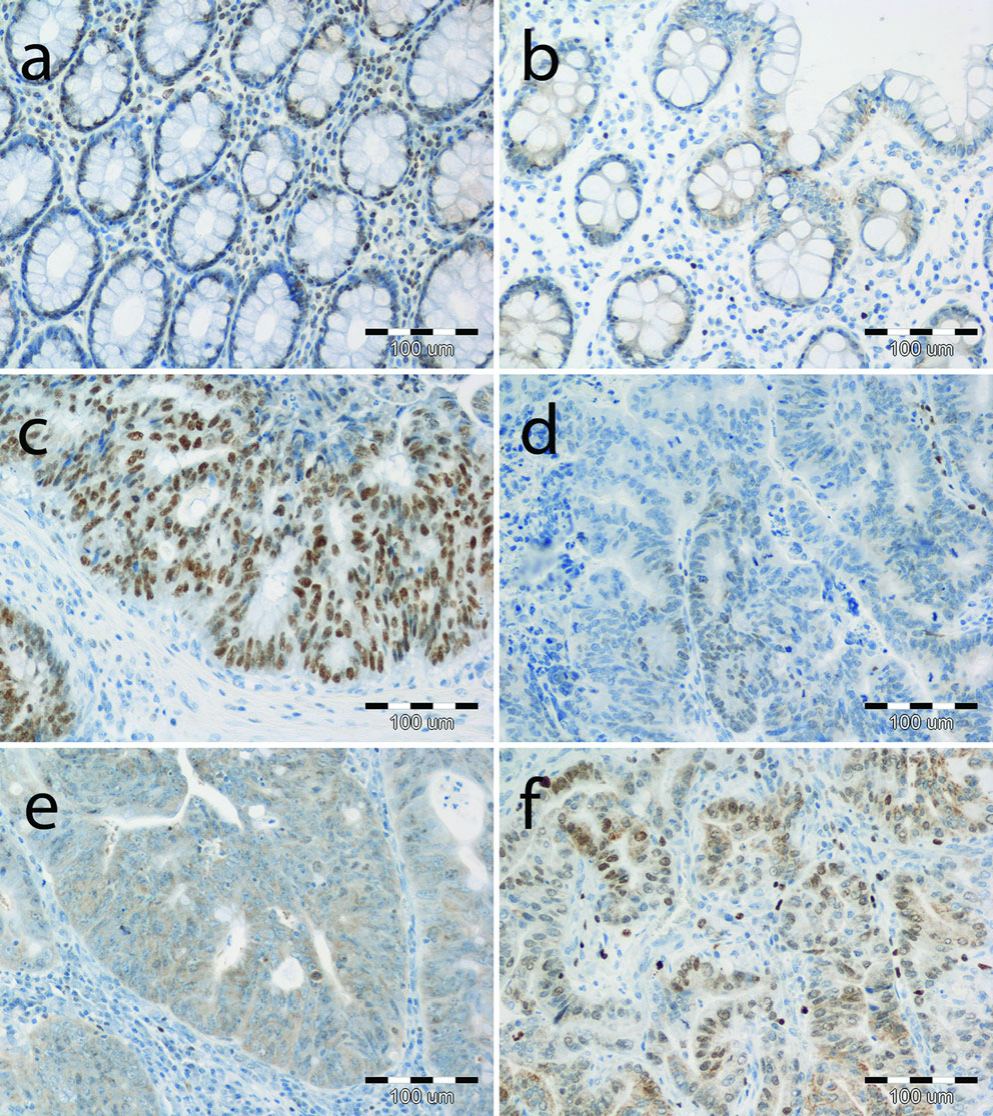
Immunohistochemical staining of SATB1 protein in representative unchanged and tumor tissues of CRC patients. **a** Nuclear and **b** dominant cytoplasmic immunoreactivity of SATB1 in unchanged colon tissue; **c** intensive nuclear, **d** low nuclear, **e** dominant cytoplasmic, and **f** combined nuclear and cytoplasmic immunoreactivity in tumor tissue. Magnification ×200

Nuclear and cytoplasmic SATB1 expression was noted in enterocytes as well as cancer cells of the analyzed tissues (Fig. 3). In addition, SATB1 immunoreactivity was shown in some stromal mononuclear cells, but they were not included in the analysis.

**Fig. 4:**
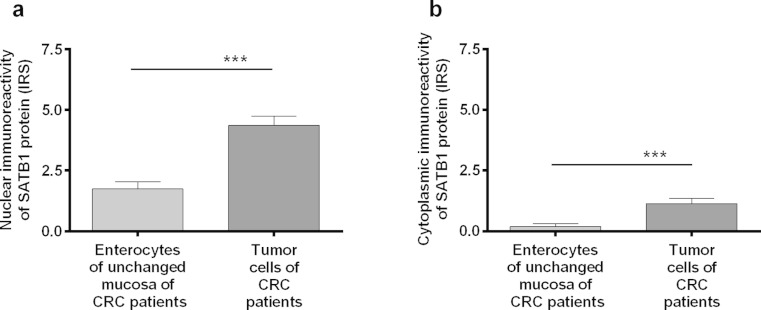
SATB1 protein expression in the tumor and unchanged tissues of the large intestine of CRC patients revealed by immunohistochemical staining. **a** Nuclear, **b** cytoplasmic immunoreactivity in enterocytes and tumor cells. Bars represent mean ± SEM. ^***^ *P* < 0.001

SATB1 nuclear expression was noted in cancer cells in 86/102 (84.3 %) of the analyzed CRC cases, whereas in enterocytes of unchanged large-intestine tissues, its expression was seen in 28/39 (71.8 %) cases. Statistical analysis revealed that the SATB1 immunoreactivity was significantly higher in cancer cells of all the analyzed CRC cases as compared to its expression noted in cells of the unchanged mucosa (IRS 4.36 ± 0.38 vs. 1.74 ± 0.31, respectively; *P* < 0.0001; Fig. 4a). Similar observations were noted when only 39 matched CRC and unchanged large intestine samples were analyzed (IRS 4.62 ± 0.62 vs. 1.74 ± 0.31; *P* < 0.0001). Cytoplasmic SATB1 expression was noted in cancer cells in 31/102 (30.4 %) CRC cases; however, unchanged large-intestine tissue enterocytes showed cytoplasmic immunoreactivity only in 3/39 (7.7 %) cases. The intensity of cytoplasmic SATB1 expression was significantly higher in cancer cells as compared to its expression in enterocytes, whether all CRC (IRS 1.14 ± 0.21 vs. 0.2 ± 0.13; *P* < 0.01; Fig. 4b) or only paired samples (IRS 1.13 ± 0.33 vs. 0.2 ± 0.13; *P* < 0.05) were analyzed. No correlation between the presence of nuclear and cytoplasmic SATB1 immunoreactivity was noted in cancer cells or enterocytes.

### The lack of correlation between SATB1 protein content and clinicopathological features

Possible correlations of SATB1 expression at the protein level with selected demographic and clinicopathological parameters were analyzed based on the results obtained by Western blotting and immunohistochemical analyses (Tables 3 and 4, respectively). SATB1 protein levels, nuclear as well as cytoplasmic immunoreactivity did not correlate with patients’ sex, age, tumor localization, TNM disease stage, depth of invasion, lymph node involvement, and the presence of metastases (Tables 3 and 4).

**Table 4:** Associations between clinicopathological features of studied CRC patients and SATB1 nuclear immunoreactivity in tumor cells

Parameter	Number of cases	Percentage (%)	SATB1 nuclear immunoreactivity in CRC tissues				
			IRS ≤ 4	Percentage (%)	IRS > 4	Percentage (%)	*P* values
Total	102	100.0	61	59.8	41	40.2	
Sex							
Male	54	52.9	34	63.0	20	37.0	0.5469
Female	48	47.1	27	56.2	21	43.8	
Age (years)							
≤67	49	48.0	29	59.2	20	40.8	1.0000
>67	53	52.0	32	60.4	21	39.6	
Localization							
Cecum, ascending, and transverse colon	37	36.3	21	56.8	16	43.2	0.8939
Descending and sigmoid colon	26	25.5	16	61.5	10	38.5	
Rectum	39	38.2	24	61.5	15	38.5	
Depth of invasion (pT status)							
T1 + T2	14	13.7	8	57.1	6	42.9	1.0000
T3 + T4	88	86.3	53	60.2	35	39.8	
Lymph nodes (pN status)							
N0	55	53.9	34	61.8	21	38.2	0.6892
N1 + N2	47	46.1	27	57.4	20	42.6	
Metastasis (pM status)							
M0	87	85.3	53	60.9	34	39.1	0.5827
M1	15	14.7	8	53.3	7	46.7	
TNM stage							
I + II	51	50.0	32	62.7	19	37.3	0.6865
III + IV	51	50.0	29	56.9	22	43.1	

### The patients’ overall survival time is not associated with the level of *SATB1* expression in CRC tissues

To assess the significance of the *SATB1* expression level as a prognostic factor, 102 patients were followed up for 36.2 months. During this observation period, 38 (37.3 %) patients died.

**Fig. 5:**
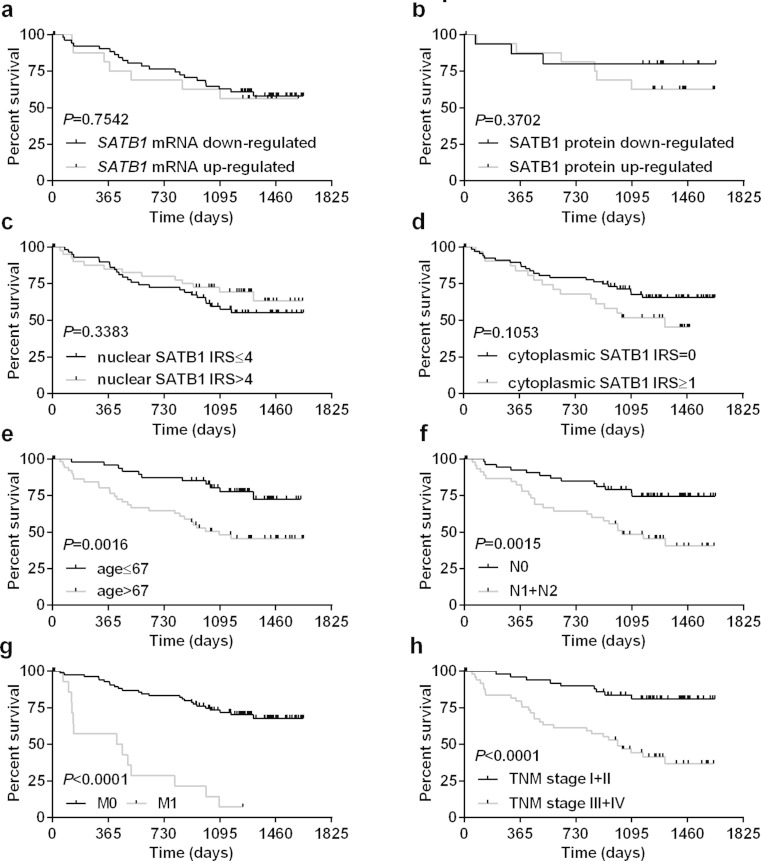
Analysis of patients’ overall survival. Kaplan-Meier survival curves of 102 CRC patients regarding **a** the expression levels of *SATB1* mRNA and **b** SATB1 protein, **c** nuclear and **d** cytoplasmic immunoreactivity of SATB1 protein, **e** patients’ age, **f** lymph node involvement, **g** the presence of metastases, and **h** TNM disease stage

The expression of *SATB1* at the mRNA and protein levels, as well as intensity of nuclear or cytoplasmic SATB1 immunoreactivity did not correlate with patients’ overall survival (Table 5; Fig. 5a–d). Of the analyzed clinicopathological and demographic parameters, advanced age at diagnosis (>67 years; *P* = 0.0016; Table 5; Fig. 5e), lymph node involvement (*P* = 0.0015; Table 5; Fig. 5f), the presence of metastases (*P* < 0.0001; Table 5; Fig. 5g), and TNM disease stage (III–IV; *P* < 0.0001; Table 5; Fig. 5h) were associated with poor patient outcome.

**Table 5:** Analysis of overall survival of CRC patients in relation to their clinicopathological characteristics and *SATB1* expression

Parameter	Deaths	/	Cases	Percentage (%)	HR	95 % CI of ratio	*P* values
SATB1 mRNA							
Downregulated	21	/	51	41.2	(1.00)	0.4748 to 2.796	0.7542
Upregulated	7	/	18	38.9	1.15		
SATB1 protein							
Downregulated	3	/	15	20.0	(1.00)	0.4920 to 6.723	0.3702
Upregulated	6	/	17	35.3	1.87		
SATB1 nuclear immunoreactivity							
Low (IRS ≤ 4)	25	/	61	41.0	(1.00)	0.3831 to 1.390	0.3383
Up (IRS > 4)	13	/	41	31.7	0.72		
SATB1 cytoplasmic immunoreactivity							
Negative (IRS = 0)	22	/	71	31.0	(1.00)	0.8869 to 3.549	0.1053
Positive (IRS ≥ 1)	16	/	31	51.6	1.69		
Sex							
Male	19	/	54	35.2	(1.00)	0.5639 to 2.013	0.8452
Female	19	/	48	39.6	1.07		
Age (years)							
≤67	11	/	49	22.4	(1.00)	1.481 to 5.310	0.0016*
>67	27	/	53	50.9	2.93		
Localization							
Cecum, ascending, and transverse colon	11	/	37	29.7	NA	NA	0.4005
Descending and sigmoid colon	11	/	26	42.3			
Rectum	16	/	39	41.0			
Depth of invasion (pT status)							
T1 + T2	3	/	14	21.4	(1.00)	0.7476 to 4.316	0.1908
T3 + T4	35	/	88	39.8	2.15		
Lymph nodes (pN status)							
N0	13	/	55	23.6	(1.00)	1.501 to 5.482	0.0015*
N1 + N2	25	/	47	53.2	2.82		
Metastasis (pM status)							
M0	25	/	87	28.7	(1.00)	15.37 to 172.5	<0.0001*
M1	13	/	15	86.7	6.44		
TNM stage							
I–II	9	/	51	17.6	(1.00)	2.104 to 7.611	<0.0001*
III–IV	29	/	51	56.9	4.33		

*Significant *P* values

## Discussion

SATB1 can globally regulate expression of many genes by tethering numerous gene loci and assembling them with chromatin remodeling enzymes and transcription factors. It has been found that SATB1 is predominantly expressed in thymocytes and regulates the spatiotemporal expression of numerous genes involved in T cell proliferation, development, and differentiation [22]. SATB1 has also been proposed as an important factor that controls the development and progression of various human neoplasms [10]; however, its role in cancer pathogenesis has yet not been fully elucidated.

The major finding of our study was the demonstration of the lower *SATB1* mRNA content in tumor tissue and unchanged mucosa of CRC patients than in the normal colon mucosa, while SATB1 protein contents in colorectal tissues of CRC patients were significantly higher than those in the colon mucosa of healthy controls. It appears that the high level of *SATB1* transcript expression in normal mucosa of healthy subjects was accompanied by relatively low SATB1 protein content. Thus, our observations suggest that in normal mucosa, posttranscriptional events limit the SATB1 expression, and this regulation becomes disturbed in colorectal carcinoma. This is a novel observation since SATB1 expression has not previously been studied in normal colon mucosa of healthy individuals, although it was determined in CRC tumor tissue [14–18].

Various mechanisms may be involved in the posttranscriptional modifications of SATB1 expression. SATB1 expression can be regulated posttranscriptionally by micro-RNAs (miRs). In breast cancer cells, miR-488 downregulated SATB1 expression, and suppression of this miR resulted in SATB1-mediated EMT [23]. Also, miR-7 and miR-155 were shown to be involved in the posttranscriptional regulation of SATB1 gene expression in breast cancer cells [24]. SATB1 expression in COLO205 colon cancer cells was downregulated by statins via posttranslational modifications of SATB1 protein which led to targeting it for proteolytic degradation [25]. Dysregulation of hypothetical SATB1 expression-controlling mechanism in colorectal tissue could lead to an aberrant SATB1 expression and result in global, tumor-promoting changes in affected cells. Further investigations, including the analysis of different epigenetic mechanisms potentially targeting SATB1 expression, are needed to delineate the differences observed between mRNA level and protein content in the studied colorectal tissues.

SATB1 expression and its role in colorectal cancer have been investigated in five studies in which samples of tumor and noncancerous colorectal tissues of CRC patients were compared; however, the colon mucosa of healthy individuals was not investigated [14–18]. It has to be noted that in none of these investigations the CRC samples were examined by the combination of three different techniques (qPCR, Western blotting, and IHC) as reported in our study. Meng et al. [14] studied only rectal cancer tissue and found a prominent heterogeneity in the expression of *SATB1* mRNA since 45/93 patients showed increased and 48/93 decreased levels of SATB1 transcripts while the average level of *SATB1* mRNA in tumor tissue was statistically higher than that in unchanged mucosa. These results are in contrast with our observations in which 81.5 % of rectal cancers had decreased levels of *SATB1* mRNA. In most cases of colorectal tumors, we found lower *SATB1* mRNA levels in comparison with unchanged colon mucosa. Completely opposite results were reported by Al-Sohaily et al. [18] who found high *SATB1* mRNA expression in 86 % of CRC tissues and lower immunoreactivity of SATB1 protein in CRC cells in comparison to adjacent noncancerous tissues. To assess the localization and levels of SATB1 protein expression in tissues of CRC patients, we also used immunohistochemical staining. We revealed nuclear and cytoplasmic immunoreactivity of the SATB1 protein in cancer cells as well as in cells of unchanged tissues; however, the nuclear expression was dominant. Our finding of higher SATB1 nuclear immunoreactivity in CRC tumor than in the matched noncancerous mucosa is in line with the results of other authors [14–17], although there are differences in the percentage of immunopositive tissues. Using only IHC, Nodin et al. [15] analyzed the expression of SATB1 protein by tissue arrays in 529 cases of CRC and 16 samples of adjacent, benign-appearing colorectal mucosa. They found that 58.0 % of the tumors were SATB1 immunonegative, and among samples of control tissues, 87.5 % were denoted as having negative expression. Fang et al. [17] observed positive immunoreactivities in CRC in 53 % of tissue samples, compared with only 10 % in the adjacent mucosa tissue cells, while Zhang et al. [16], in 58.8 and 2.5 %, respectively. We have noted nuclear SATB1 expression in 84.3 % of CRC tissues and 71.8 % of noncancerous mucosa of the large intestine. These discrepancies may partly result from methodological differences, such as the type of antibody used or a way of estimating of immunoreactivity; however, it is difficult to find an explanation for the divergent results of Al-Sohaily et al. [18].

In addition to nuclear SATB1 immunoreactivity, we also observed that SATB1 protein was present in the cytoplasm of cancer cells of 30.4 % studied CRC patients. Nakayama et al. [26] demonstrated that the localization of SATB1 protein in the cytoplasm could be attributed to the single point mutation within the N-terminal nuclear targeting determinant of SATB1. Further studies are needed to elucidate whether the elevated cytoplasmic expression of SATB1 in CRC tumors in comparison to unchanged tissues is a result of mutations and if there is an association between mutations in *SATB1* gene and impaired SATB1 intracellular targeting.

To assess SATB1 expression in tumor and unchanged mucosa at the protein level, we used also Western blotting method. So far, this method has been used by other authors only to study SATB1 expression in cell lines. SATB1 protein content was the highest in tumor tissues; however, in contrast to the results of the immunohistochemical analysis, there was no statistically significant difference in comparison with the unchanged tissues. This discrepancy could be due to the fact that the results obtained by Western blotting method may be obscured by the presence of mononuclear cell infiltration in analyzed tissues.

In the present study, the level of SATB1 expression in CRC tissues did not correlate with patients’ sex, age, tumor localization, TNM disease stage, depth of invasion, lymph node involvement, and the presence of metastases; furthermore, it had no impact on patients’ overall survival. Previous studies provided contradictory data about prognostic value of SATB1 expression. Meng et al. [14] revealed a positive correlation of SATB1 overexpression with the progression of human rectal cancer. They found that SATB1 expression correlated with invasive depth and TNM stage at both protein (assessed by IHC) and mRNA levels. Correlations between mentioned parameters and SATB1 expression were also reported by Zhang et al. [16]. In the tissue microarray study, SATB1 overexpression assessed by IHC alone failed to be a reliable prognostic marker; however, a strong, significant association was found between SATB1 expression and female gender, microsatellite stability, β-catenin overexpression, and SATB2 expression, but not with any other conventional clinicopathological parameters [15]. On the contrary, Al-Sohaily et al. [18] observed that loss of SATB1 expression in the analyzed cohort of 352 CRC patients was an unfavorable prognostic factor. However, ours and the latter study significantly differed in the median follow-up period which was nearly 36 months in our study and 66 months in the other one [18].

In summary, *SATB1* mRNA and SATB1 protein levels in tumor tissues differed significantly from those observed in the normal mucosa; thus, it may be assumed that altered SATB1 expression can precede the CRC-associated lesions. Better understanding of the mechanisms leading to the alterations in the control of SATB1 expression in precancerous colorectal lesions and CRC tissue may provide deeper insight into the pathogenesis of colorectal cancer.

## References

[1] Ferlay J, Shin HR, Bray F, Forman D, Mathers C, Parkin DM (2010). Estimates of worldwide burden of cancer in 2008: GLOBOCAN 2008. Int J Cancer.

[2] Pavan Kumar P, Purbey PK, Sinha CK, Notani D, Limaye A, Jayani RS (2006). Phosphorylation of SATB1, a global gene regulator, acts as a molecular switch regulating its transcriptional activity in vivo. Mol Cell.

[3] Han HJ, Russo J, Kohwi Y, Kohwi-Shigematsu T (2008). SATB1 reprogrammes gene expression to promote breast tumour growth and metastasis. Nature.

[4] Kobierzycki C, Wojnar A, Dziegiel P (2013). Expression of SATB1 protein in the ductal breast carcinoma tissue microarrays—preliminary study. Folia Histochem Cytobiol.

[5] Zhao XD, Ji WY, Zhang W, He LX, Yang J, Liang HJ (2010). Overexpression of SATB1 in laryngeal squamous cell carcinoma. ORL J Otorhinolaryngol Relat Spec.

[6] Sun F, Lu X, Li H, Peng Z, Wu K, Wang G (2012). Special AT-rich sequence binding protein 1 regulates the multidrug resistance and invasion of human gastric cancer cells. Oncol Lett.

[7] Tu W, Luo M, Wang Z, Yan W, Xia Y, Deng H (2012). Upregulation of SATB1 promotes tumor growth and metastasis in liver cancer. Liver Int.

[8] Xiang J, Zhou L, Li S, Xi X, Zhang J, Wang Y (2012). AT-rich sequence binding protein 1: contribution to tumor progression and metastasis of human ovarian carcinoma. Oncol Lett.

[9] Li QQ, Chen ZQ, Xu JD, Cao XX, Chen Q, Liu XP (2010). Overexpression and involvement of special AT-rich sequence binding protein 1 in multidrug resistance in human breast carcinoma cells. Cancer Sci.

[10] Kohwi-Shigematsu T, Poterlowicz K, Ordinario E, Han HJ, Botchkarev VA, Kohwi Y (2013). Genome organizing function of SATB1 in tumor progression. Semin Cancer Biol.

[11] Iorns E, Hnatyszyn HJ, Seo P, Clarke J, Ward T, Lippman M (2010). The role of SATB1 in breast cancer pathogenesis. J Natl Cancer Inst.

[12] Selinger CI, Cooper WA, Al-Sohaily S, Mladenova DN, Pangon L, Kennedy CW (2011). Loss of special AT-rich binding protein 1 expression is a marker of poor survival in lung cancer. J Thorac Oncol.

[13] Zhou LY, Liu F, Tong J, Chen QQ, Zhang FW (2009). Expression of special AT-rich sequence-binding protein mRNA and its clinicopathological significance in non-small cell lung cancer. Nan Fang Yi Ke Da Xue Xue Bao.

[14] Meng WJ, Yan H, Zhou B, Zhang W, Kong XH, Wang R (2012). Correlation of SATB1 overexpression with the progression of human rectal cancer. Int J Color Dis.

[15] Nodin B, Johannesson H, Wangefjord S, O’Connor DP, Ericson-Lindquist K, Uhlén M (2012). Molecular correlates and prognostic significance of SATB1 expression in colorectal cancer. Diagn Pathol.

[16] Zhang J, Zhang B, Zhang X, Sun Y, Wei X, McNutt MA (2013). SATB1 expression is associated with biologic behavior in colorectal carcinoma in vitro and in vivo. PLoS One.

[17] Fang XF, Hou ZB, Dai XZ, Chen C, Ge J, Shen H (2013). Special AT-rich sequence-binding protein 1 promotes cell growth and metastasis in colorectal cancer. World J Gastroenterol.

[18] Al-Sohaily S, Henderson C, Selinger C, Pangon L, Segelov E, Kohonen-Corish M (2014). Loss of special AT-rich sequence-binding protein 1 (SATB1) predicts poor survival in patients with colorectal cancer. Histopathology.

[19] Livak KJ, Schmittgen TD (2001). Analysis of relative gene expression data using real-time quantitative PCR and the 2(-Delta Delta C(T)) Method. Methods.

[20] Bradford MM (1976). A rapid and sensitive method for the quantitation of microgram quantities of protein utilizing the principle of protein-dye binding. Anal Biochem.

[21] Remmele W, Stegner HE (1987). Recommendation for uniform definition of an immunoreactive score (IRS) for immunohistochemical estrogen receptor detection (ER-ICA) in breast cancer. Pathologe.

[22] Alvarez JD, Yasui DH, Niida H, Joh T, Loh DY, Kohwi-Shigematsu T (2000). The MAR-binding protein SATB1 orchestrates temporal and spatial expression of multiple genes during T-cell development. Genes Dev.

[23] Li QQ, Chen ZQ, Cao XX, Xu JD, Xu JW, Chen YY (2011). Involvement of NF-κB/miR-448 regulatory feedback loop in chemotherapy-induced epithelial-mesenchymal transition of breast cancer cells. Cell Death Differ.

[24] McInnes N, Sadlon TJ, Brown CY, Pederson S, Beyer M, Schultze JL (2012). FOXP3 and FOXP3-regulated microRNAs suppress SATB1 in breast cancer cells. Oncogene.

[25] Lakshminarayana Reddy CN, Vyjayanti VN, Notani D, Galande S, Kotamraju S (2010). Down-regulation of the global regulator SATB1 by statins in COLO205 colon cancer cells. Mol Med Rep.

[26] Nakayama Y, Mian IS, Kohwi-Shigematsu T, Ogawa T (2005). A nuclear targeting determinant for SATB1, a genome organizer in the T cell lineage. Cell Cycle.

